# Primary left ventricular leiomyosarcoma: a case report

**DOI:** 10.1186/s13019-024-02680-4

**Published:** 2024-04-02

**Authors:** Vipin Balachandran, Vanessa Young, Tina Baillie, Allen James

**Affiliations:** 1 Conjoint Associate Lecturer, School of Medicine and Public Health, University of Newcastle; Adjunct Teaching Fellow, University of Western Australia, Perth, Australia; 2 Douglass Hanly Moir Pathology, Newcastle, Australia; 3 Department of Cardiothoracic Surgery, John Hunter Hospital, Ward F3, Lookout Road, New Lambton Heights, Newcastle, NSW 2305 Australia

**Keywords:** Leiomyosarcoma, Cardiac tumour, Left ventricular tumour

## Abstract

Cardiac leiomyosarcomas are a rare subset of the already infrequent, primary malignant cardiac neoplasia spectrum. The most common site for a primary leiomyosarcoma of the ventricle is on the right with fewer than five globally reported cases in the left ventricle. Most present with non-specific symptoms but attention is usually sought after the appearance of compressive symptoms or arrhythmias. We present a case of a left ventricular leiomyosarcoma in a 50-year old female patient that had a delayed diagnosis and its subsequent surgical resection and oncological management with docetaxel and gemcitabine. This case highlights the need for a high index of suspicion for cardiac masses especially if there are competing chronic diseases with similar symptomatology. Given the rare presentation of left ventricular leiomyosarcomas, case reports may provide valuable information that is otherwise unavailable.

**Supplementary Information:**

The online version contains supplementary material available at 10.1186/s13019-024-02680-4.

## Background

A primary cardiac neoplasm is a rare diagnosis with a reported prevalence of between 0.001 and 0.03% in autopsy series. The most frequent malignant primary tumours are angiosarcomas with the rarest being leiomyosarcomas [1]. The location of malignant neoplasms also vary with angiosarcomas being commonly found in the right atrium and intimal sarcomas such as osteosarcomas and leiomyosarcomas arising from the left [2]. We present a case of an extremely rare primary cardiac leiomyosarcoma arising from the left ventricle which was surgically managed in the first instance.

## Case report

**Fig. 1:**
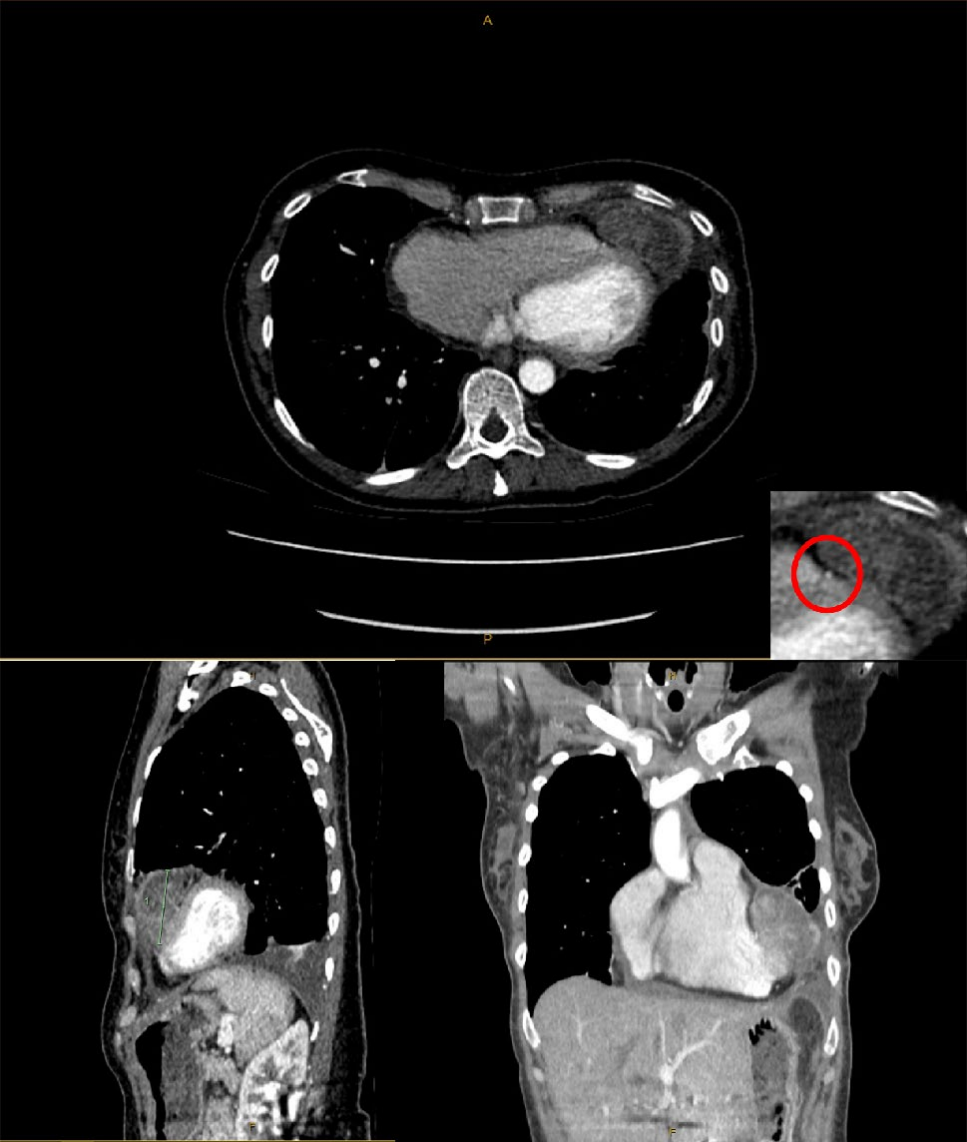
Composite image of CT with arterial phase contrast. Top: Axial view showing tumour arising from apex of left ventricle. Inset: image showing tumour arising lateral to the LAD. Bottom left: Sagittal view. Bottom right: Coronal view. Note that this was initially reported as a loculated intra-pericardial collection

A 50 year old female patient with a background of systemic lupus erythematosus presented to various hospitals with chest pain and shortness of breath over three years without any clear diagnosis. The history was also significant for sweats, low grade temperatures and weight loss of seven kilograms. A computerised tomograph (CT) of the chest was notable as having described a loculated intrapericardial effusion causing compression of the left ventricle (Fig. 1). Unfortunately, despite multiple imaging attempts, a diagnosis of a tumour was not made until recently when a trans-thoracic echocardiogram (TTE) showed the presence of a mass either arising from the pericardium or ventricle. A left video assisted thoracoscopic (VAT) pericardial window and biopsy of the mass was done which was reported as inflammatory tissue without evidence of malignancy.

A re-presentation with heart failure prompted a trans-oesophageal echocardiogram which demonstrated a mass compressing the left ventricle. A coronary angiogram was normal apart from feeding vessels into the tumour. Due to the multiple hospital admissions and new heart failure, a decision was made to excise the tumour.

**Fig. 2:**
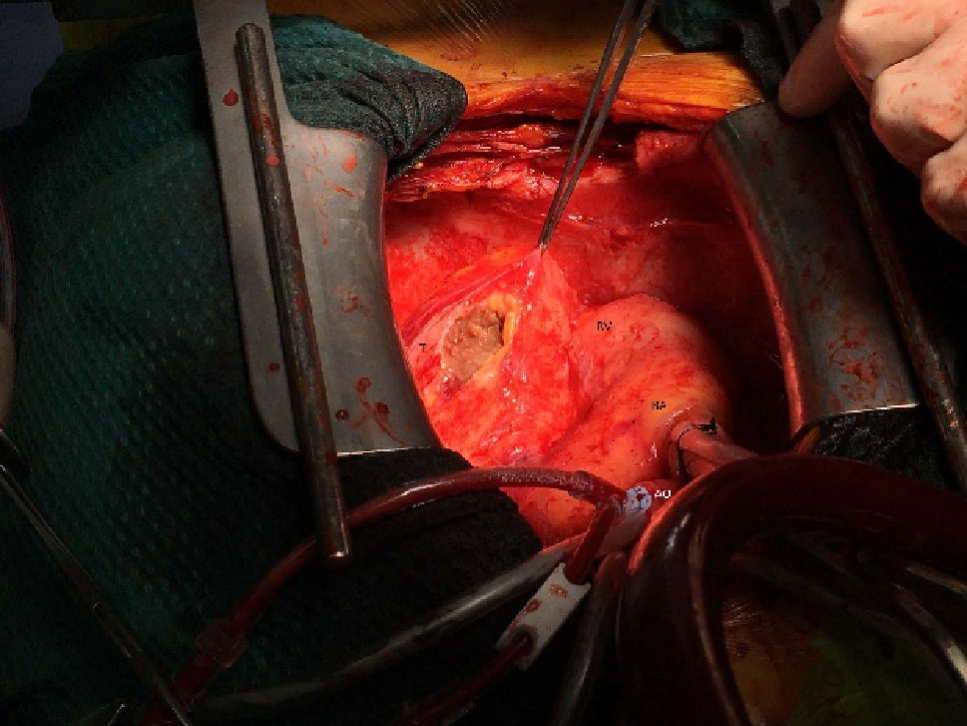
Intraoperative specimen. Key: Ao – aorta, T - tumour, RA - right atrium, RV – right ventricle

A median sternotomy was performed and the patient placed on cardiopulmonary bypass cannulating the aorta and right atrium. A large mass arising from the apex of left ventricle was identified arising immediately lateral to the left anterior descending coronary artery and was carefully mobilised (Fig. 2a) Cardioplegic arrest allowed a full thickness ventriculotomy and subsequent removal of the tumour. The defect was repaired with a continuous 2 − 0 Prolene stitch followed by overlocking 4 − 0 Prolene with a Teflon buttress. The phrenic nerves were spared bilaterally. The patient was easily weaned off cardiopulmonary bypass but had to have the chest packed due to generalised coagulopathy. The chest was unpacked and closed primarily the following day after which she made an uneventful post-operative recovery.

A follow up TTE demonstrated a preserved left ventricular cavity size and systolic function (ejection fraction 70%) with apical akinesis presumably from where the tumour was removed.

**Fig. 3:**
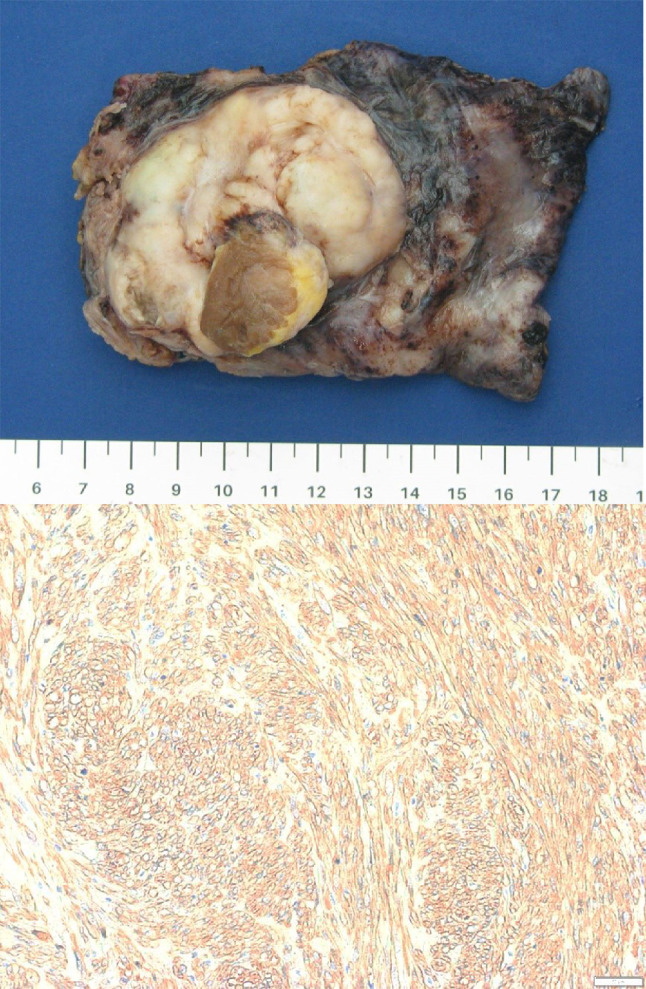
Composite pathology imaging. Top: Gross specimen. Bottom: Expression of Smooth muscle actin (200x)

Gross examination of the pathology revealed a 65 × 45 × 32 mm nodular mass attached to a portion of cardiac muscle (Fig. 3). The cut surface showed an infiltrative solid white lesion containing areas of necrosis and haemorrhage. Histologic examination revealed a pleomorphic spindle cell tumour with heterogenous, lobular architecture, widely invading the myocardium. The constituent tumour cells were spindled and epithelioid, arranged in diffuse sheets and interlacing fascicles within a background of variably myxoid stroma. A spectrum of nuclear atypia was seen, including moderately pleomorphic blunt nuclei with vesicular chromatin transitioning to areas with marked cytologic atypia. Frequent mitotic figures were identified, numbering up to 32 per 10 high power fields. Broad zones of necrosis were present and lymphovascular invasion was identified.

Immunohistochemistry revealed the tumour cells to be positive for smooth muscle actin and desmin (Fig. 3), with only focal positive staining for pan-cytokeratin and AE1/AE3. The tumour cells were negative for CK7, CK20, EMA, myogenin, MYO D1, STAT 6, calretinin, CD31, CD34, ERG, SOX 10, S100 and p40.

The morphologic features and immunohistochemical profile were in keeping with a high grade cardiac leiomyosarcoma.

Three cycles of docetaxel and gemcitabine chemotherapy was started by the treating oncology team after discharge from hospital. At the last surgical review in September 2020, the patient was well and fast approaching the one year mark from the initial surgery. PET scans performed until December 2021 did not pick up recurrence or metastasis but a CT scan performed in December 2021 picked up metastasis to the right lung along with a probably recurrence at the suture line. This was followed by evidence of metastatic disease to the neck in 2023. The patient passed away in late 2023 with a recurrence of the tumour in her lungs and concomitant heart failure.

## Discussion

Primary cardiac tumours are exceedingly rare neoplasms comprising less than 1% of the 0.001–0.28% of all reported cardiac malignancies. The left atrium is the most common site reported in literature with the left ventricle being the rarest cardiac site [2–4]. Primary left ventricular leiomyosarcomas have not been well described in literature due to its rarity. Our case is also notable that it differs from previously published cases where the tumour was predominantly inside the left ventricular cavity whereas our case had a mass causing extrinsic LV compression [4–7]. A case report presented by Khalid et al. at the American Heart Association 2014 Scientific Sessions and Resuscitation Science Symposium bears similarities to our own with regards to the tumour location but unfortunately does not detail the peri-operative course [8]. 

Diagnosis has been reported to be straightforward with echocardiography, CT and MRI having very high sensitivity [4]. Unfortunately as is highlighted by our case, vague symptoms with the differential diagnosis confounded by the presence of chronic disease can lead the clinician into ordering imaging that does not focus on the heart. In a particularly unfortunate turn of events in this case, a biopsy of the mass via VAT returned inflammatory tissue. Regardless, we recommend the use of echocardiography and CT to aid in the diagnosis if vague cardiac symptoms are reported.

Wang et al. reported that surgery was the most common therapy provided followed by adjuvant chemotherapy. Incomplete resection was a feature of many of these cases due to the invasive nature of the tumour. This was also a feature of our case as the aim was for macroscopic resection of the tumour without aiming for strict neoplastic resection margins which would have left the patient with very little functioning ventricle.

Prognosis has been universally reported as poor with Wang et al. noting that the five year survival rate was 25.4%. Adjuvant chemotherapy was also noted to improve outcomes in many studies [3, 4]. 

### Electronic supplementary material

Below is the link to the electronic supplementary material.

Supplementary Material 1

Supplementary Material 2

Supplementary Material 3

Supplementary Material 4

Supplementary Material 5

Supplementary Material 6

## Data Availability

Additional photomicrographs can be obtained from application to the corresponding author.

## Abbreviations

CT: Computerised Tomography
MRI: Magnetic Resonance Imaging
TTE: Transthoracic Echocardiogram
VAT: Video Assisted Thoracoscopy
