# Supplementary figures and images for: Primary left ventricular leiomyosarcoma: a case report
Source: J Cardiothorac Surg. 2024 Apr 2;19:168. doi: 10.1186/s13019-024-02680-4 (PMC10985868; doi:10.1186/s13019-024-02680-4)

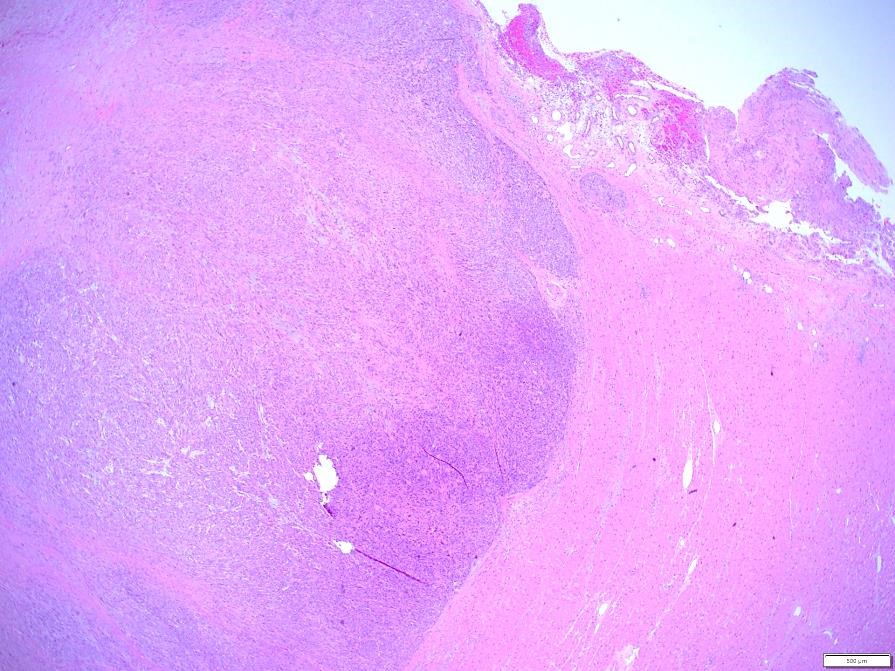

Supplement: Supplementary file 1 — Supplementary Material 1 [file 13019_2024_2680_MOESM1_ESM.jpg]

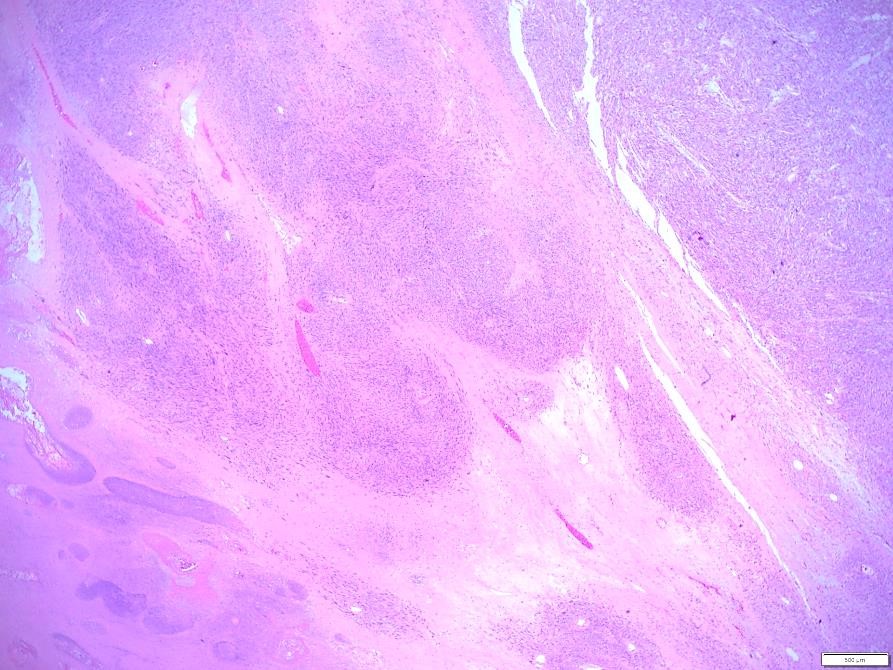

Supplement: Supplementary file 2 — Supplementary Material 2 [file 13019_2024_2680_MOESM2_ESM.jpg]

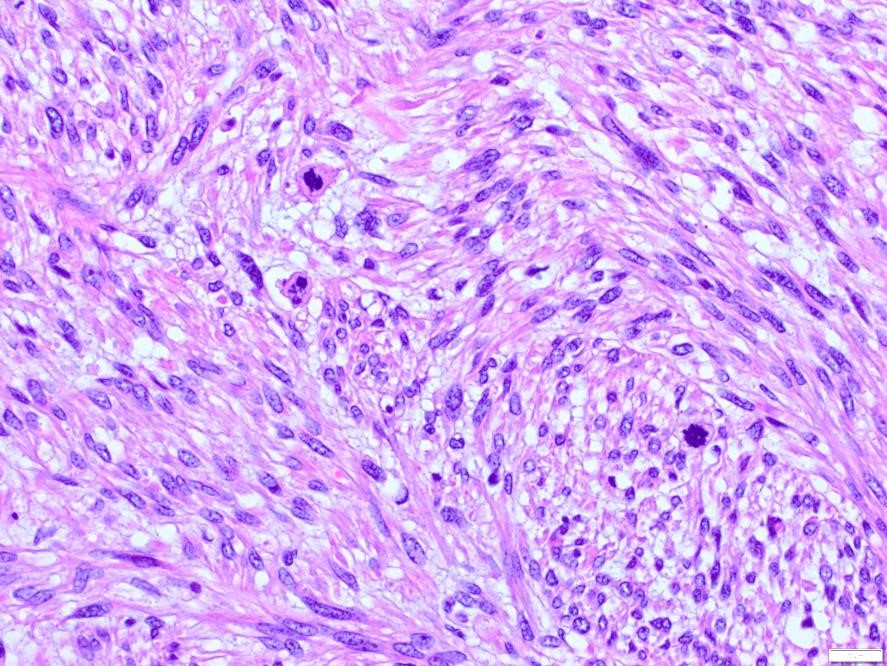

Supplement: Supplementary file 3 — Supplementary Material 3 [file 13019_2024_2680_MOESM3_ESM.jpg]

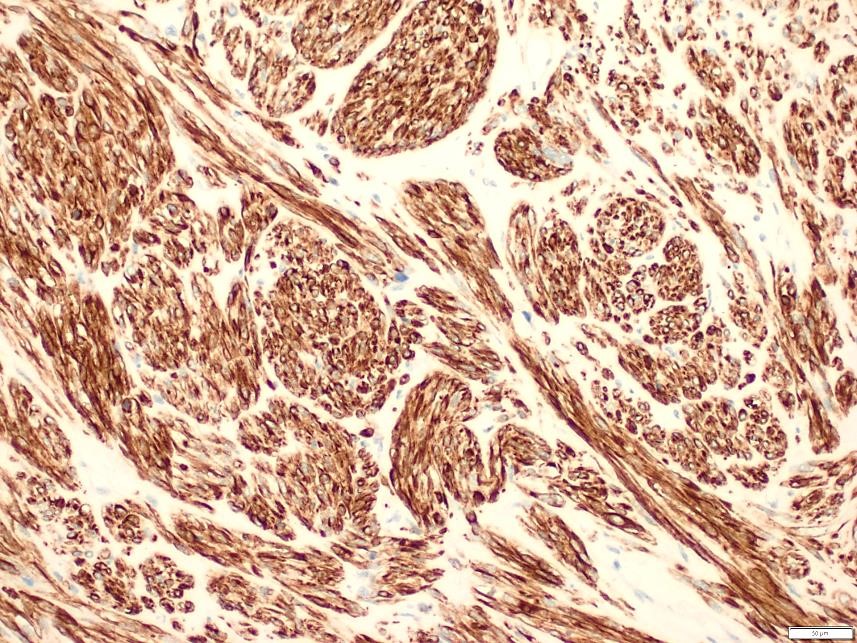

Supplement: Supplementary file 4 — Supplementary Material 4 [file 13019_2024_2680_MOESM4_ESM.jpg]

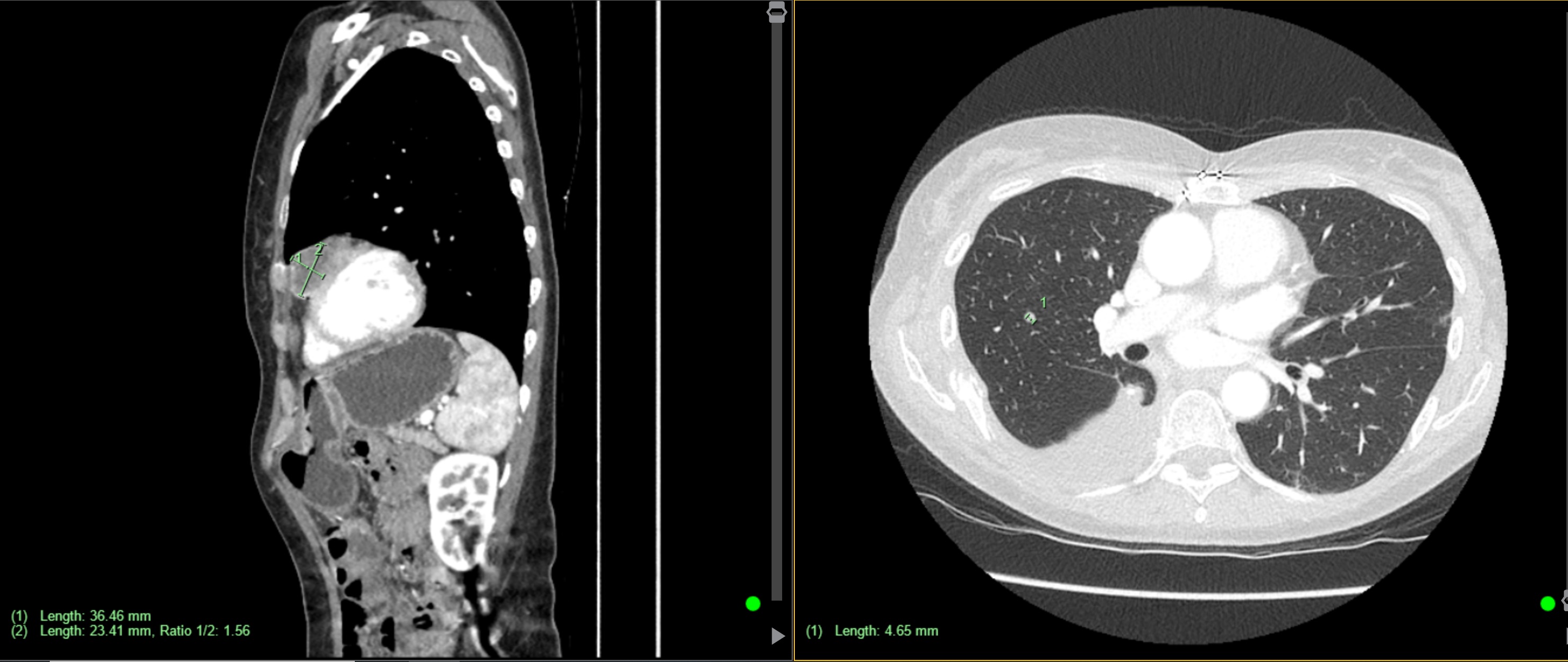

Supplement: Supplementary file 5 — Supplementary Material 5 [file 13019_2024_2680_MOESM5_ESM.jpg]

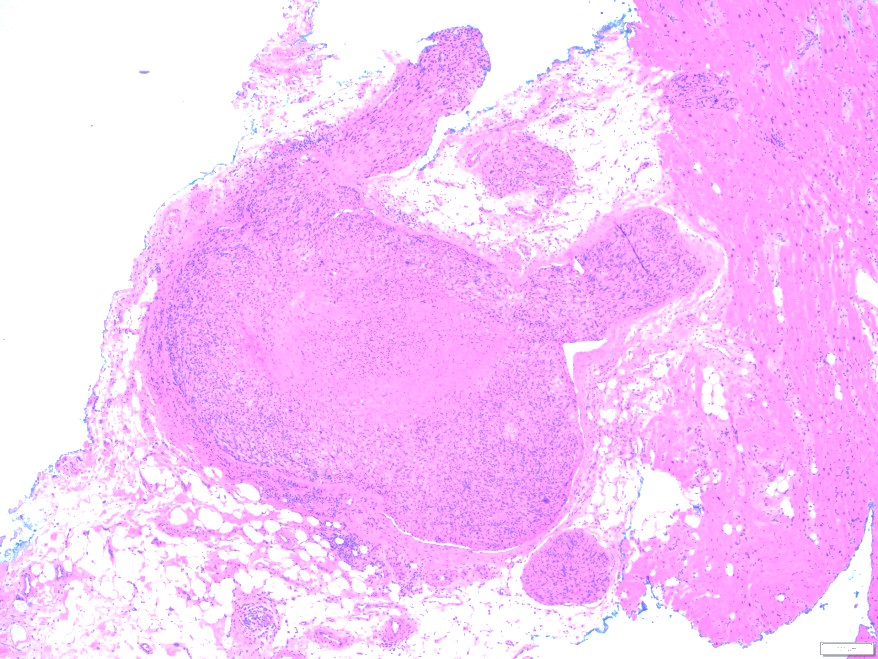

Supplement: Supplementary file 6 — Supplementary Material 6 [file 13019_2024_2680_MOESM6_ESM.jpg]
